# A flashing success: A community-engaged approach to finding western U.S. fireflies

**DOI:** 10.1371/journal.pone.0341617

**Published:** 2026-07-29

**Authors:** Christy Bills, Gavin Martin, Ellen Eiriksson, Arthur Morris, Alexandra G. Duffy, Seth M. Bybee

**Affiliations:** 1 Natural History Museum of Utah, University of Utah, Salt Lake City, Utah, United States of America; 2 School of Arts and Sciences, Laramie County Community College, Cheyenne, Wyoming, United States of America; 3 Adaptive Stewardship, Conservation, and Ecological Restoration, Salt Lake City, Utah, United States of America; 4 Department of Biological Sciences, North Carolina State University, Raleigh, North Carolina, United States of America; 5 Department of Biology and Monte L. Bean Museum, Brigham Young University, Provo, Utah, United States of America; Instituto Federal de Educacao Ciencia e Tecnologia Goiano - Campus Urutai, BRAZIL

## Abstract

Fireflies are well-documented in many regions of the USA. However, the presence of flashing fireflies in much of the western United States remains largely unknown. Leveraging citizen science, this study aimed to locate populations of bioluminescent fireflies across Utah. Through a multifaceted public outreach campaign and an interactive website for data collection, citizen scientists were invited to report firefly sightings. Over nearly a decade, the project successfully amassed reports from diverse locations, significantly expanding the known distribution of bioluminescent fireflies in Utah and beyond. Validation of reports was conducted through on-site observations and specimen collection. Despite challenges in data reports (e.g., reports from outside desired area) and public interest management (e.g., protecting private property sites), the project achieved remarkable success, with over 135 unique localities documented. Media coverage, social media engagement, and educational outreach further amplified the impact of the project. Limitations in data quality and public engagement were addressed through iterative improvements in reporting protocols and outreach strategies. Future directions include expanding the project to encompass the western United States and exploring innovative communication strategies. By partnering with organizations in neighboring states, the project aims to create a robust dataset and foster public awareness of these charismatic invertebrates. This study highlights the effectiveness of community-engaged approaches in biodiversity research and underscores the importance of public involvement in scientific endeavors.

## Introduction

Fireflies (Lampyridae) are a common family of beetles (Coleoptera) found around the world with >2600 described species [1,2]. They are famous for their bioluminescent displays as adults that are usually associated with courtship, although not all species are bioluminescent as adults [href:https://paperpile.com/c/k930PR/jkLwi] [3]. In Utah, fireflies that do not bioluminesce are relatively well known. However, it is not common knowledge that there are flashing fireflies in Utah.

From natural history collection data, flashing fireflies have been documented in Utah for over a hundred years. A female *Pyractomena dispersa* was collected next to Utah Lake by E. P. Van Duzee on June 25th 1922 [4]. Since 1922, a handful of other researchers have documented populations of *P. dispersa* in northern Utah. For example, in 1991 a specimen was caught in Roosevelt Utah as part of a competition to find bioluminescence in Utah [5]. Another three examples include personal accounts from Northeastern Utah that were reported to the Natural History Museum of Utah (NHMU), but none of these were authoritatively documented or confirmed. These occurrences combined with the work of Green [5] made for less than 10 known localities for flashing fireflies in Utah, but only those of Green [5] were documented and even most of these did not have discrete label data (e.g., lacking dates of occurrence or locality information beyond a city). Infrequent, unverified reports of scattered populations of bioluminescent fireflies are intriguing; they motivate questions related to population and community ecology, particularly in the context of the expansion of human influence on the western landscape and climate change.

From anecdotal reports, Utah bioluminescent fireflies inhabit marshy areas. However, little is known about how widespread populations are throughout the state. Further, we do not know if populations are expanding or contracting, nor whether isolated populations are persisting. In light of other imperiled firefly populations in North America [6] there is an urgency to gathering this information. A critical step to addressing these questions is to locate populations of fireflies across Utah, an accomplishment that has largely eluded academic and professional entomologists. We leveraged the power of community-engaged science to identify localities of *P. dispersa* throughout the state. We report here on our initial efforts to enlist citizen scientists across a large area and some of the lessons we have learned. Lessons from our efforts apply to other large-scale community science efforts.

## Materials and methods

In an attempt to radically expand possibilities for detecting bioluminescent fireflies, we began to explore avenues that involved public input from across Utah in 2014. Our approach aimed to increase the number of verifiable occurrence records across the state of Utah during the brief temporal window in which bioluminescent fireflies flash. The temporal window for fireflies is an approximate six-week activity period during early May through early July depending on spring temperatures, precipitation and elevation.

The project consisted of two parts: a public information campaign to inform members of the public about the project and a website to receive data reports.

The public information campaign included press releases, radio, television and podcast interviews, talks given by researchers involved in the project at rural libraries and nature centers, blog posts, paid ads on social media, exhibits at our host museums, and mailings throughout the state where we surmised the appropriate audience would see them. The website was designed with in-house technical assistance at NHMU and made to be as simple as possible to not deter data reporters. We refined it over time as we learned the way members of the public interacted with it. For example, we created pull-down menus for county names to make it easier for data reporters.

### A path forward and timeline

In the spring of 2012, we were notified of a large population in Spanish Fork, Utah. We collected specimens from this population and confirmed they were identified as *P. dispersa*. A local news agency was at the site while our team was there to collect specimens; they ran a story on the nightly news. A follow-up news story was done by Brigham Young University (BYU) in June 2013. Subsequently, we began to get reports of firefly sightings throughout the state via email and telephone. We realized the opportunity to track firefly populations with reports from enthusiastic citizen scientists and formulated a plan to do so in a more formal way than email and telephone.

In the Spring of 2014, the NHMU launched a webpage (https://nhmu.utah.edu/fireflies) to allow community scientists, referred to as “data reporters,” to learn about the project and report recent and historical sightings. Starting in 2015, NHMU began a three-pronged marketing approach to get the word out: 1) traditional media, 2) social media, and 3) in-person outreach. This approach aimed to increase the number of reports across a wide area during the brief temporal window in which bioluminescent fireflies flash in Utah.

#### Traditional media.

**Fig 1:**
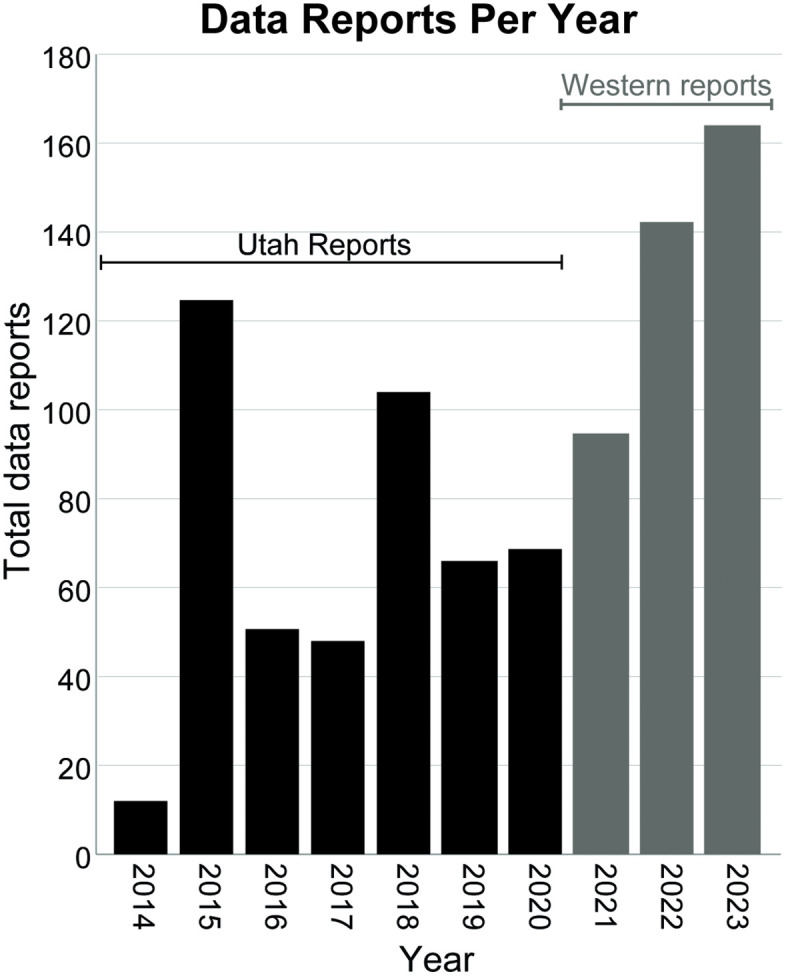
Firefly locality reports starting in 2014 to the present. Black bars (2014-2020) represent reports only for Utah fireflies. Starting in 2021 the project was expanded to include firefly reports from western states (gray bars). These gray bars represent reports from both Utah and other western (e.g., Nevada, Colorado, New Mexico, Idaho, etc.).

Once the website was in place, several local TV stations and news agencies ran stories. The University of Utah and BYU media also published stories and videos about the firefly project (S1 Table). Additionally, we mailed postcards and brochures to campgrounds, state parks and recreation supply stores for posting. Community scientists immediately began making reports using the website (Fig 1). Similar numbers of postcards and brochures were distributed in subsequent years by mail and handed out at the museum’s outreach events around the state. In 2017, a press release was sent to rural newspapers advertising the project and requesting participation.

#### Social media.

Social media engagement occurred both purposefully and organically. We used targeted Facebook ads in 2018 as another way to engage social media. These ads were aimed at counties where we had limited firefly reports and were seeking more data. Meanwhile, the public began generating social media connections and YouTube videos that helped to publicize the project and likely motivated more reports of firefly sightings.

#### Validation of reports.

After receiving a report it was evaluated by museum staff. For unique, reliable reports, trained volunteers or museum staff, or researchers (collectors) were sent to validate locations by also observing the fireflies and collecting specimens. Validation was difficult for several reasons: even when we knew where to look in the general area, the fireflies often occupied areas of only a few hundred square meters, so they were easy to miss, and the fireflies flashed poorly or not at all in high winds or temperatures cooler than about 60 degrees F (which often occurred even during the summer at high elevations). When flashing fireflies were located by the project representative, and collecting was feasible (i.e., the environment and population size permitted), 5–10 fireflies were collected. These specimens were either deposited in a −80 C freezer (BYU Monte L. Bean Museum Cryo Insect Collection) or if deposited at NHMU pinned or in 70% ethanol. Specimens stored at NHMU are databased at https://ecdysis.org/.

## Results

Response to our firefly project has been widespread and enthusiastic. In 2013, just before community science engagement began, we had less than 10 documented locations of bioluminescent fireflies. In 2014, after we soft launched the website, we received 12 unique reports. Since 2015, report submissions have continued to come in. At the end of the 2023 season, we had a total of 859 reports with 92.7% credibility, resulting in a total of 796 useful data points. In total, we have now amassed approximately 135 unique localities for fireflies in Utah. Data points represent 27 of the 29 counties in Utah. Specimens from 19 of Utah’s counties were also collected and deposited in the Natural History Museum of Utah. Additionally, we currently have reports from surrounding states (Colorado, Idaho, Montana, Nevada, New Mexico, and Wyoming) and even into British Columbia, Canada.

In total, we have had 459,936 visits to the original Western Firefly Project map hosted on Google. Media attention was hard to calculate but a YouTube video (*Nature’s fireworks: glowing fireflies lighting up Utah*) focused on our project has garnered more than 403,000 views. Social media efforts resulted in 8806 engagements (7046 reactions and 1760 shares) that we could trace. Other notable results from this effort include a children’s book (*The Mystery of Luci’s Missing Lantern*) and several exhibits around the state encouraging citizen science and firefly conservation. One particular exhibit is a traveling exhibit that has been to multiple nature centers throughout Utah. It is difficult to quantify the impact of the traditional media (TV news, newspapers, magazines, etc.). However, when looking at their overall viewership a conservative estimate is that the efforts of this project reached between 750,000–1,500,000 individuals from TV news reports and written media. The year 2015 was the most popular year for reports (>120) in the state of Utah. After we expanded the project to neighboring states in the Intermountain West from 2021–2023 the number of reports went up even more (Fig 1).

## Discussion

Using a citizen science approach, we were able to rapidly identify over a hundred populations of bioluminescent fireflies; initially just in Utah but later throughout the intermountain West. By asking for, and facilitating community input, we were able to find firefly populations in widely scattered locations across a broad area. This approach helped to overcome several challenges in locating firefly populations that made it difficult for a small team of researchers. First, Intermountain western firefly populations are only active for a brief period, approximately six weeks (personal observation of the authors), so there is a relatively short window of time to observe them. Secondly, western bioluminescent firefly populations are highly localized, scattered throughout rural communities and wildlands, in non-recreative areas. Thirdly, firefly activity can be suppressed even during peak season due to weather conditions such as wind and cool temperatures; this is especially a problem at higher elevations where temperatures can dip below activity thresholds.

Another objective of the project was to invite members of the non-research community into the data-gathering process. Citizen science engagement is valuable in connecting public audiences to scientific research [7]. Members of the non-research community were successfully invited into the science process, by our direct efforts and by viral communications between others. Fireflies are unique and the public is generally positive towards these animals [8]. Thus, public interest was relatively easy to generate. Talks have been well attended when hosted in person or online. Participants in the project, when met with a collecting volunteer or researcher, were eager to share in the process.

As so many people from such a wide set of backgrounds became involved, we experienced many of the challenges reported by others for data quality in community science [9,10]. We found that some of those challenges appear to be intractable and part of the tradeoff that’s necessary for the benefit of a broad set of observers [11]. However, it appears that some of the community science challenges we experienced can probably be remedied.

### Limitations and remedies

Data reported by the public has inherent pitfalls. To remedy the possible inclusion of erroneous data, we combined common-sense evaluations of reports with evaluations based on information from scientists familiar with firefly natural history. It took a large amount of labor to assess each data report for accuracy, but it was necessary. The vast majority of reports appeared reliable and were found to be credible, however, approximately 7% of data reports were not included in our data set because they did not appear to represent actual bioluminescent firefly populations, based on the location (seen in a dense urban area) or the time of day they were spotted (9 am in traffic, for example) or an unusual description (multi-color, blinking at 3 am in a high dry desert). Further, other reports were evaluated as non-valid because they were not plausible (e.g., submitted a copy of a photo of a Japanese firefly, out of season or diel pattern, completely incorrect habitat, etc.) or because they did not report enough information to visit the locality or at least contact the data reporter.

Re-locating reported populations was difficult in the field even when GPS locations had been reported. To remedy the difficulty of finding reported populations, we invited the people who made reports to meet museum representatives in the field. We found that site visits were most effective when the original data reporter was willing to meet a project representative. Data reports that fell into a gray area were followed up with emails to the data reporter, which were answered in about a third of cases.

It was difficult to obtain accurate location reports. To remedy location-reporting errors, we improved reporting forms and instructions. Originally, we simply asked where fireflies had been seen. This was unclear for many reporters as they were not aware of their exact locality. We added a pull-down menu for which state the fireflies were seen in, and another field for county. We also requested a GPS location. People began reporting GPS locations that were not for the firefly populations, but could have been – and in some cases were – for the location of the observer or at the home address of the observer. Based on the variety of answers we received, it became clear that more specific reporting options and directions were needed to improve the data. For example, we added the question: “Is the GPS mark you provided your location or the exact firefly location?” As of 2022, in addition to drop-down menu fields for state and county, we added point mapping in a more familiar format, similar to Google Maps, for exact firefly locations. Further, we could have done a better job of educating data reporters about what we are looking for in terms of data points (the exact locality of the fireflies) and data from their location (e.g., what does the firefly habitat look like) so that they can provide more nuanced data.

### Public interest and data ethics

A difficulty faced with the original design of the data reporting form was an open map that exactly showed the reported firefly localities. To remedy misuse of the map, we changed the map to mask exact population locations and clearly asked people to respect firefly locations. We found the general public used the data as a “firefly tourism” guide, especially at a few locations. Although we continue to work towards transparency with the data we have been provided, there is a need to be respectful to private landowners and small communities that are not equipped or tolerant of nighttime eco-tourism. In 2021, we chose to change the map to suggest a general area where fireflies had been reported, and direct people to not visit sites without permission. Firefly tourism is on the rise significantly [12,13], especially post-COVID [14–16]. We hope to create a balance between sharing the data appropriately and protecting sites, private property and small communities from over-visitation.

**Fig 2:**
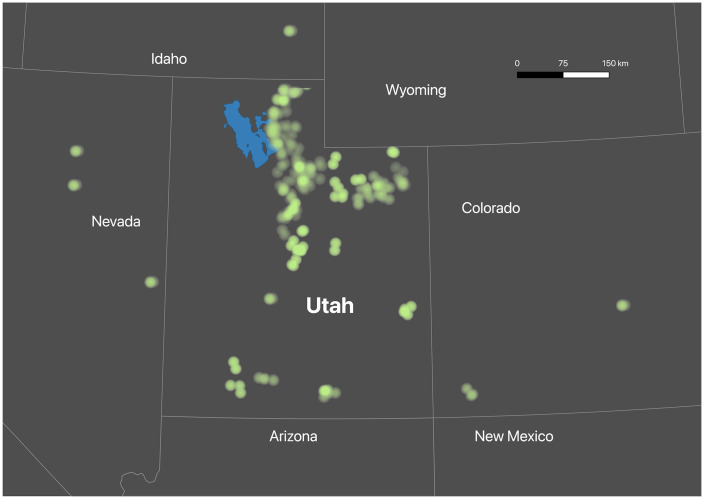
Heatmap of firefly locality reports for Utah and other western states. In total >130 localities throughout Utah and surrounding states are shown using QGIS version 3.44.8. To protect participant privacy, private lands, and potentially threatened populations, we aggregated sightings using a DBSCAN clustering algorithm with a minimum cluster size of two and maximum distance of 20 km. We then filtered out isolated, non-clustered sightings before running a kernel density estimation (KDE) with a quartic shape and 10 km radius. Map features, including state boundaries and the Great Salt Lake depicted in blue, were derived from Natural Earth public domain. Reports for the states surrounding Utah began in 2021 and will likely increase in the future.

Once locations were masked on the map, some uses of the data became difficult. To remedy problems of unnecessary location masking, we provide information on request. We continue to make a heat map of localities publicly available (Fig 2), and a map of specific localities available to researchers, upon request, with justification. We suggest individuals and field trip groups only visit dedicated viewing areas (e.g., Nibley, Utah where there is a firefly park dedicated to firefly conservation). We also share information about firefly conservation (e.g., light pollution) with schools and on our website.

### Reaching People

It was naturally a challenge to reach people, especially in some of the relatively remote, rural areas where firefly populations exist. To remedy challenges of reaching people, we used a mixed model of both “physical” and “digital” approaches for public outreach to reach participants. We did not specifically assess the success of each approach. Each has its strength, however, digital approaches appear to have had the most reach.

#### Physical.

**Fig 3:**
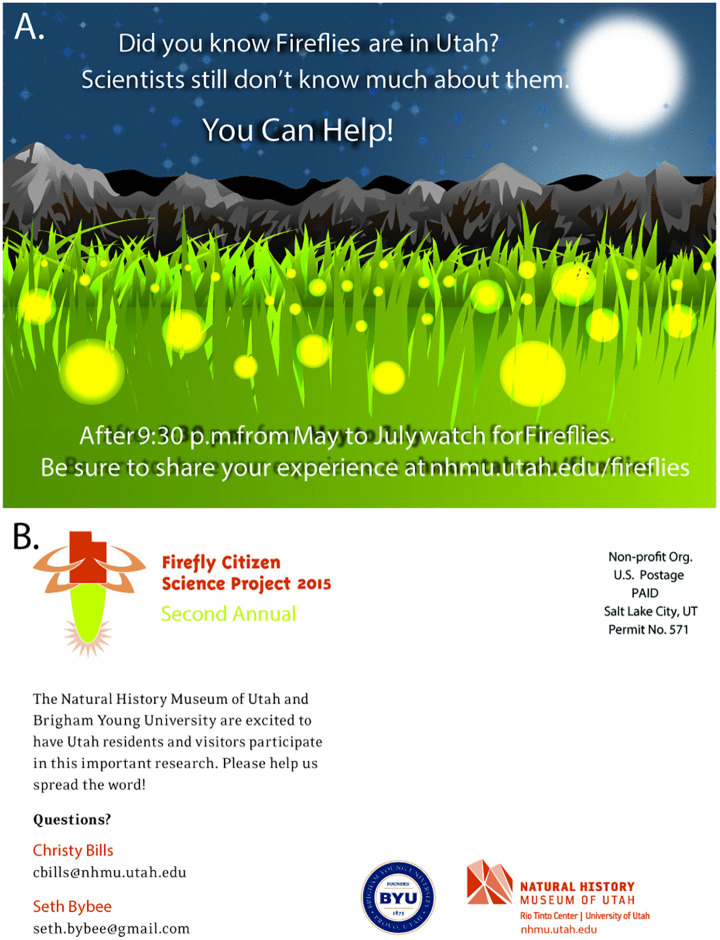
Example postcards sent throughout the state. A.) Front of the postcard. B.) Back of the postcard.

*Postcards:* Creating paper postcards (Fig 3) and mailing them around the state was labor-intensive but not very cost-intensive. One benefit of the postcards was that they remained available (e.g., on bulletin boards and in windows) in locations for years as a physical reminder for the project.

*Public outreach events:* Giving talks in libraries, nature centers and small rural museums is also relatively labor intensive but it has a strong impact. It appeared to us that impact was increased when attendees asked questions about the project. We found that although the attendees of those events were usually already excited about natural history topics, many had not heard of citizen science (judged by a raise of hands). We think that visiting their communities was worth the effort. Although visiting communities may not have yielded a large number of reports, it did yield reports from areas where we had little to no information. Additionally, and most importantly, we were able to provide value for these communities that are often underserved, making the project less consumptive of community knowledge and more of an exchange.

*Exhibits:* Additionally, NHMU created exhibit material that promoted the project. Within a larger exhibit about the local natural world, “*Nature All Around Us.*” This project was highlighted, complete with a large-scale, light-up firefly and videos interviewing local participants and researchers. A similar exhibit was done independently at the local zoo. A smaller exhibit was made that could travel to other natural history museums to promote fireflies in Utah and the project. We count these as energy-intensive yet long-lasting high-impact exchange efforts.

*Press releases:* We sent out press releases in 2017 and 2018 (S1 Fig) to both urban and rural news outlets. We were particularly interested in the rural audience since these populations were thought to potentially have the most knowledge about fireflies. The press releases did not yield large amounts of reporting from these communities for the project, however, they may have yielded important localities for data coverage.

#### Digital.

*Electronic Media advertisement:* We used museum listservs, Facebook, Instagram and X as a way to engage potential reporters through social media. Over the course of the project, Facebook’s popularity among younger users waned while Instagram and X platforms gained in younger users [17]. The impact of emails sent to museum members was hard to quantify. The marketing team at NHMU used targeted Facebook ads to encourage residents of particular counties where our project lacked data to participate. The marketing team also used Instagram and X to encourage participation in the project. X had the least amount of engagement among the platforms we used accounting for less than 3% of engagements.

*Podcast:* We have done podcasts with national audiences that do get archived on the internet. It is unclear how much podcasts seem to drive locals to participate in our project. The same is true for interviews with regional publications and local public radio interviews. They create a small, immediately impactful digital footprint but likely lack a long-term impact.

*Institutional advertisements:* The most impactful digital impact seemed to be short videos put together and advertised by our home institutions. These received more views than local TV spots, advertisements or published news. For example, in 2014 Brigham Young University made a short, under two-minute YouTube video describing these fireflies and our research. With over 403,000 views for this one video and thousands more from others, our institutional advertisements appear to be the most impactful advertising done for the project. Interestingly the impact of these advertisements does not appear to have been felt until the following year, 2015. This is likely because these advertisements were posted in July 2014 just before the July Fourth holiday. This was a few weeks after the firefly season of 2014.

### Future directions

As we move to the next phase of this project by expanding outside of Utah and into the western United States, we will need to cast an even larger net to have the success we’ve accumulated thus far. We have considered including social media influencers who already have a big audience, outside the usual audiences from our institutions. In addition to podcasts, finding weekly or bi-weekly spots on either TV or radio shows during peak firefly season (May-June) with hosts that would be interested in talking to us about firefly-related topics generally (e.g., what they are, life cycle, what they eat, conservation, light pollution, etc.), and firefly related activities (where to see and learn about them) that benefit our project, specifically. Additionally, gamifying our process for reporting through a kind of contest, where there are rewards (e.g., passes to our museums and/or behind-the-scenes tours) for the most credible reports. We are still grappling with the need for such efforts as a broader net may dilute our scientific efforts with much more spurious data to sift through, when the data we have now is sound and largely satisfies the scientific questions we seek to answer.

## Conclusions

The inpouring of reports has led to us expanding the project from the Utah Firefly Project to the Western Firefly Project. We now partner with organizations in other states including The Albuquerque Biopark, the Butterfly Pavilion in Westminster Colorado, and the Missoula Butterfly House in Montana. They will seek firefly data in their area and encourage local participants to input data into our web form. This optimizes resources and allows us to work together to make a more robust data set. We continue to engage the public and improve methods for that engagement and for increasing the quality of reports, verifying reports, and sampling populations.

After almost a decade of working on this project and trying to attract citizen scientists from throughout the state, most people still do not know that there are bioluminescent adult fireflies in Utah. We still have work to do. However, our project has been highly successful, though we acknowledge that not all projects have such a charismatic organism. We have demonstrated that this type of work can be done at a high level when the public is excited about the project and focal organism. We are ready to take on the next stage of the project– the western US.

## Supporting information

S1 Fig Example press releases. In 2017 and 2018 we released press releases to create awareness of the project.
(PDF)

S1 Table Firefly Press 2013–2022. Media coverage of the project is provided and organzied by year. Links to the media are provided along with the title.
(PDF)

## References

[1] Martin GJ, Stanger-Hall KF, Branham MA, Da Silveira LFL, Lower SE, Hall DW, et al. Higher-level phylogeny and reclassification of lampyridae (Coleoptera: Elateroidea). Insect Systematics and Diversity. 2019;3(6). doi: 10.1093/isd/ixz024

[2] Ferreira VS, Keller O, Branham MA. Multilocus phylogeny support the nonbioluminescent fireflychespiritoas a new subfamily in the lampyridae (Coleoptera: Elateroidea). Insect Systematics and Diversity. 2020;4(6). doi: 10.1093/isd/ixaa014

[3] Martin GJ, Branham MA, Whiting MF, Bybee SM. Total evidence phylogeny and the evolution of adult bioluminescence in fireflies (Coleoptera: Lampyridae). Mol Phylogenet Evol. 2017;107:564–75. doi: 10.1016/j.ympev.2016.12.017 27998815

[4] Green JW. Revision of the nearctic species of pyractomena (Coleoptera: Lampyridae). The Wasmann Journal of Biology. 1957;15:1–20.

[5] Wilson A. Firefly find helps children see science in new light. The Salt Lake Tribune. 1991.

[6] Fallon CE, Walker AC, Lewis S, Cicero J, Faust L, Heckscher CM, et al. Evaluating firefly extinction risk: Initial red list assessments for North America. PLoS One. 2021;16(11):e0259379. doi: 10.1371/journal.pone.0259379 34788329 PMC8598072

[7] Dickinson JL, Shirk J, Bonter D, Bonney R, Crain RL, Martin J, et al. The current state of citizen science as a tool for ecological research and public engagement. Frontiers in Ecol & Environ. 2012;10(6):291–7. doi: 10.1890/110236

[8] Ho JZ, Wu CH, Chen YH, Yang PS. New trend of ecological industry—as example of value and development of firefly watching activities in Mt. Ali area. Formosan Entomologist. 2009.

[9] Crall AW, Newman GJ, Stohlgren TJ, Holfelder KA, Graham J, Waller DM. Assessing citizen science data quality: An invasive species case study. Conservation Letters. 2011;4(6):433–42. doi: 10.1111/j.1755-263x.2011.00196.x

[10] Swanson A, Kosmala M, Lintott C, Packer C. A generalized approach for producing, quantifying, and validating citizen science data from wildlife images. Conserv Biol. 2016;30(3):520–31. doi: 10.1111/cobi.12695 27111678 PMC4999033

[11] Anhalt-Depies C, Stenglein JL, Zuckerberg B, Townsend PA, Rissman AR. Tradeoffs and tools for data quality, privacy, transparency, and trust in citizen science. Biological Conservation. 2019;238:108195. doi: 10.1016/j.biocon.2019.108195

[12] Thancharoen A (2012) Well managed firefly tourism: A good tool for firefly conservation in Thailand. Lampyrid 2:142–8.

[13] Ayub S, Mohd Rosli NA, Abdullah M, Sulaiman N. Assessment of firefly abundance at a new ecotourism site of the Bernam river, Selangor, Peninsular Malaysia. Serangga. 2017;22:33–46.

[14] Lemelin R, Jaramillo-López P, López-Ocaña N, Del-Val E. In the still of the night: Firefly tourism in Mexico. Anatolia. 2020;32(1):12–22. doi: 10.1080/13032917.2020.1819832

[15] Cheng S, Faidi MA, Tan S-A, Vijayanathan J, Malek MA, Bahashim B, et al. Fireflies in Southeast Asia: Knowledge gaps, entomotourism and conservation. Biodivers Conserv. 2021;30(4):925–44. doi: 10.1007/s10531-021-02129-3

[16] Chow AT, Chong JH, Cook M, White D. Vanishing fireflies: A citizen-science project promoting scientific inquiry and environmental stewardship. Science Education and Civic Engagement. 2014;6(1):23–31.

[17] Ortutay B, Associated P. Facebook fights for relevance as Gen Z turns away: ‘I don’t even remember the last time I logged in’. Fortune. 2023.

