# Supplementary material for: A flashing success: A community-engaged approach to finding western U.S. fireflies
Source: PLoS One. 2026 Jul 29;21(7):e0341617. doi: 10.1371/journal.pone.0341617 (PMC13421758; doi:10.1371/journal.pone.0341617)
Supplement: S1 Fig — In 2017 and 2018 we released press releases to create awareness of the project. (PDF) [file pone.0341617.s001.pdf]

NATURAL HISTORY
MUSEUM OF UTAH

NEWS RELEASE
www.newsdesk.nhmu.utah.edu

Kristine Chapman
Public Relations Assistant
Natural History Museum of Utah
kchapman@umnh.utah.edu
Office: 801.585.9142

Christy Bills
Invertebrate Collections Manager
Natural History Museum of Utah
cbills@nhmu.utah.edu
Office: 801.587.7907

Researchers Benefit from Crowd Sourcing and Citizen Science Volunteers

Searching for firefly habitats in Utah

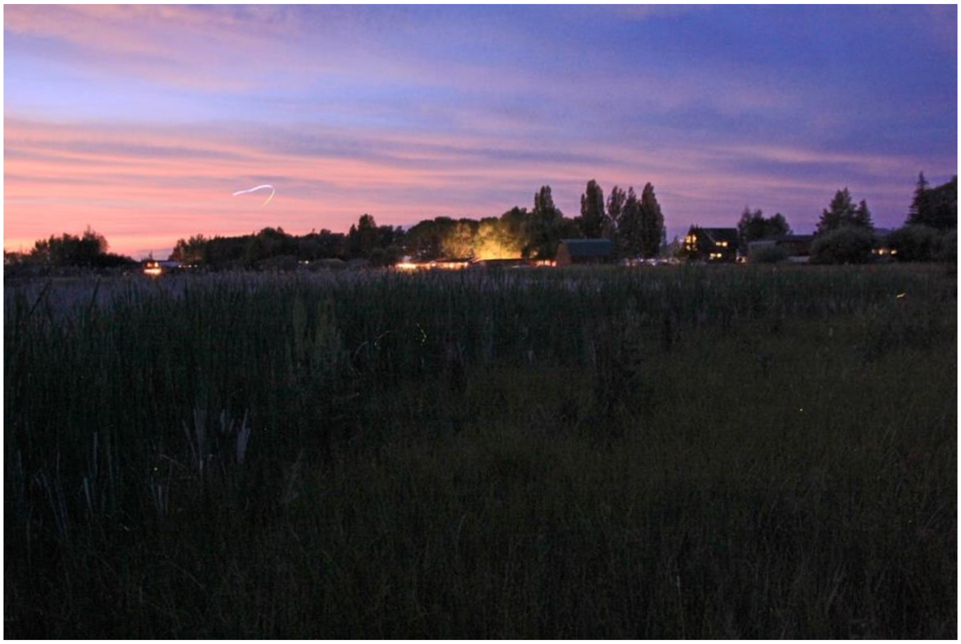

Faint light of fireflies beginning to appear – Bear Lake Valley

The warm glow of a firefly is one of nature's most endearing and mystical sights, and local scientists are once again asking Utahn's across the state to act as local researchers by sharing information about firefly sightings on a data collection website.

"Firefly mating season is about to begin, and we are hoping to enlist nature lovers across the state to be our eyes and ears and help us gather information about firefly sightings and habitat locations," says Christy Bills, Entomology Collections Manager at the Natural History Museum of Utah. Bills and researchers from Brigham Young University have joined forces to study firefly populations throughout Utah.

To help get the word out, the Museum has even designed a new mini firefly exhibit highlighting the details and purposes of the joint study, a few interesting facts about fireflies, the types of habitats they thrive in and how vital citizen participation can be in a state-wide science project such as this.

As surprising as it may seem, fireflies are actually bioluminescent beetles and are not a new phenomenon in the state. Researchers believe they have been here a long time, and since the oldest firefly specimen housed at the Museum was collected in 1929, the evidence supports that conclusion. Even in an extremely dry climate like Utah's, lightning bugs are attracted to area wetlands, swampy spaces and damp rural pastures away from human-created light sources. It is in these conditions where fireflies choose to mate, where females deposit their eggs and where the larvae will feed underground for up to two years before the new generation starts the ritual all over again.

"Last year, we were at Bear Lake a little earlier than usual," said seasonal resident Nate Brunson, "and we decided to take a walk along the park boardwalk through the wetlands to the beach. As soon as we stepped out into the marsh, about 10 feet in the air just below the horizon, it looked like Tinkerbell and a thousand of her cousins were going crazy sprinkling pixie dust. It was a magical light show of fireflies for almost 5 minutes before it died down to only a few random blinks. It was stunning and so amazing, and I hope we can experience something similar again this year!"

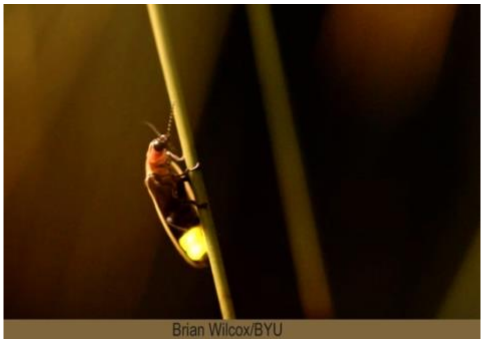

Firefly encounters like this have happened to quite a few unsuspecting Utahns and it's not too late to become a citizen scientist and learn more about how to be involved. Join Christy Bills at the Natural History Museum of Utah on Wednesday, May 23, 2018 – 6pm for a presentation about her research and ways to participate in the Citizen Science Firefly Project. Browse the Museum website for additional information: https://nhmu.utah.edu/fireflies.

Additional Resources

- Video by BYU researchers about fireflies in Utah: https://youtu.be/JFOmPQWw4Io
- Report firefly sightings: https://nhmu.utah.edu/utah-firefly-sighting-submission-form
- Firefly facts: https://nhmu.utah.edu/blog/2016/06/28/fierce-facts-about-fireflies
- Firefly story with audio: http://upr.org/post/tiny-spark-firefly-gone-viral

###

About the Natural History Museum of Utah

The Natural History Museum of Utah at the University of Utah is a premier scientific research and cultural institution. It opened to the public in 1969 and moved into a spectacular, award-winning new home in 2011 at the Rio Tinto Center in Salt Lake City. The Museum's 30 scientists oversee active field research programs throughout Utah and elsewhere and help care for natural history collections of more 1.6 million objects. The Museum offers innovative exhibitions and educational programs to thousands of residents and visitors each year, including timely and interactive temporary and permanent exhibits, numerous special events and other programs. The Museum reaches 450,000 people annually on-site and in communities and classrooms statewide.
