# Supplementary material for: A flashing success: A community-engaged approach to finding western U.S. fireflies
Source: PLoS One. 2026 Jul 29;21(7):e0341617. doi: 10.1371/journal.pone.0341617 (PMC13421758; doi:10.1371/journal.pone.0341617)
Supplement: S1 Table — Media coverage of the project is provided and organzied by year. Links to the media are provided along with the title. (PDF) [file pone.0341617.s002.pdf]

| | | reactions | shares | total |
|---|---|---|---|---|
| 2014 | | | | |
| | 16 May, FB, promoting project (18 comments, 117 shares, 154 rxns) - NHMU | 154 | 117 | 271 |
| | 13 July, FB, promoting project (15 comments, 168 shares, 80 rxns) - NHMU | 80 | 168 | 248 |
| | 17 July, FB, sharing NatGeo ff article and project (2 comments 5 shares, 50 rxns) | 50 | 2 | 52 |
| 2015 | | | | 0 |
| | 29 April, FB, sharing KSL story about project, (81 comments, 270 shares, 854 rxns) - NHMU | 854 | 270 | 1124 |
| | 17 Jun, Twitter, sharing Catalyst article about project (3 likes, 1 repost) - NHMU | 3 | 1 | |
| | 13 July, FB, sharing Fox13 story about project, (69 comments, 189 shares, 519 rxns) - NHMU | 519 | 189 | 708 |
| | 14 July, Twitter, sharing project, Fox13 article (no reactions) | | | |
| | 18 Aug, FB, sharing High Country News article about project (1 comment, 18 shares, 146 rxns) - | 146 | 18 | 164 |
| | 13 July, Twitter, sharing project, tagged BYU, (5 likes, 3 reposts) - NHMU | 5 | 3 | |
| | | | | 0 |
| 2016 | 6 June, FB, promoting project (107 comments, 1.1K rxns) | 1100 | na | 1100 |
| | 8 June, FB, promoting citizen science, FF project mentioned (3 comments, 289 rxns) | 289 | na | 289 |
| | 21 June, FB, promoting blog about project (3 comments, 28 shares, 143 rxns) | 143 | 28 | 171 |
| | 2 July, FB, promoting firefly facts blog (4 comments, 27 shares, 191 rxns) - NHMU | 191 | 27 | 218 |
| | 31 Aug, Twitter, promoting project (no reactions) | | | |
| | 3 Sept, Twitter, promoting project (1 like) -NHMU | 1 | | |
| 2017 | | | | 0 |
| | 16 June, FB, promoting project (133 comments, 263 shares, 797 rxns) | 797 | 263 | 1060 |
| | 17 July, FB live video discussing entomology and project (23 rxns, 8 shares) - NHMU | 23 | 8 | 31 |
| 2018 | | | | 0 |
| | 20 April, FB, promoting firefly exhibit - NHMU (47 comments, 103 shares) | na | 103 | 103 |
| | April, NHMU exhibit | | | 0 |
| | 3 May, KCPW, interview | | | 0 |
| | 7 May, FB promoting NHMU firefly talk - (8 rxns) - NHMU | 8 | 0 | |
| | 21 May, FB, sharing Standard Examiner article abour project (28 comments, 22 shares, 65 rxns) | 65 | 22 | 87 |
| | 23 May, FB, promoting NHMU presentation (30 rxns) | 30 | | 30 |
| | 23 May, Twitter, promoting NHMU presentation (32 likes, 10 shares) | 32 | 10 | |
| | 23 May NHMU, presentation - CB | na | na | na |
| | 31 May, FB, promoting project, (69 comments, 138 shares, 420 rxns) - NHMU | 420 | 138 | 558 |
| | 7 June, Twitter, promoting project (16 likes, 2 reposts) - NHMU | 16 | 2 | |
| | 10 Jun, FB, sharing Herald article about Ut fireflies (17 comments, 27 shares, 148 rxns) - NHMU | 148 | 27 | 175 |

| | | | | | |
|---|---|---|---|---|---|
| | | 19 Jun, Twitter, promoting firefly exhibit (2 likes, 2 reposts) - NHMU | 2 | 2 | |
| ? | | 20 Jun, FB, mentioned in NHMU research update (8 comments, 13 shares, 143 rxns) | 143 | 13 | 156 |
| | | 10 Aug, Twitter, promoting creation of firefly exhibit (5 likes) -NHMU | 5 | 0 | |
| | | 15 Aug, FB, promoting creation of firefly exhibit at NHMU (4 comments, 3 shares, 140 rxns) | 140 | 3 | 143 |
| | | 8 Oct, FB, promoting firefly presentation @NHMU (3 shares, 36 comments) | | 3 | 3 |
| | | 9 Oct, Twitter, promoting firefly presentation at NHMU (4 likes, 1 repost) - NHMU | 4 | 1 | |
| | | 10 Oct firefly presentation at NHMU - CB | na | na | na |
| | 2019 | | | | 0 |
| | | 23 Feb Utah State Univ, presentation - CB | na | na | na |
| | | 23 April FB, sharing nature.org blog, (36 comments, 101 shares, 444 rxns) - NHMU | 444 | 101 | 545 |
| | | 15 May, FB, promoting project, (13 rxns) | 13 | na | 13 |
| | | 15 May, Twitter, promoting project (2 likes, 7 reposts) - NHMU | 2 | 7 | |
| | | 17 May, FB promoting project (4 comments, 11 rxns) | 11 | 4 | 15 |
| | | 22 May FB promoting project, (2 comments, 5 shares, 20 rxns) - NHMU | 20 | 5 | 25 |
| | | 22 May Twitter, promoting project (1 like, 2 reposts) - NHMU | 1 | 2 | |
| | | 31 May FB promoting project (12 comments, 25 shares, 81 rxns) - NHMU | 81 | 25 | 106 |
| | | 31 May Twitter, promoting project with link (7 likes, 4 shares) - NHMU | 7 | 4 | |
| | | 2 Jun, FB, promoting blog about project (71 comments, 54 shares, 180 rxns) - NHMU | 180 | 54 | 234 |
| | | 5 June, Twitter, promoting the project (5 likes, 1 repost) - NHMU | 5 | 1 | |
| | | 12 June, Twitter, promoting project (4 likes, 1 reposts) - NHMU | 4 | 1 | |
| | | 19 June, Twitter, promotion project (2 likes) - NHMU | 2 | 0 | |
| | | 26 Jun, Twitter, sharing Daily Herald article (5 likes 2 repost) | 5 | 2 | |
| | | 26 Jun, sharing Daily Herald article about project (1 comment, 26 rxns) | 26 | na | na |
| | | 1 July, Twitter, promoting project, inc Idaho (4 likes, 1 repost) | 4 | 1 | |
| | 2020 | | | | 0 |
| | | | | | 0 |
| | | 28 April Swaner Nature Center, presentation- CB | na | na | na |
| | | 2 March Ogden Nature Ctr, presentation - CB | na | na | na |
| | | 12 May, FB, promoting project, (1 comment, 3 shares, 14 rxns) | 14 | 3 | 17 |
| | | 21 May, Twitter, promoting project (4 likes, 1 repost) | 4 | 1 | |
| | | 27 May, Twitter, promoting project, (8 likes, 4 reposts) | 8 | 4 | |
| | | 29 May, Twitter, promoting project (2 likes, 1 repost) | 2 | 1 | |
| | | 3 June, FB, promoting project, (30 comments, 54 shares, 389 rxns) | 389 | 54 | 443 |

| | | | | |
|---|---|---|---|---|
| | 10 June, Twitter, sharing NHMU Youtube video of Charleston fireflies - (6 likes, 1 repost) | 6 | 3 | |
| | 16 June, Twitter, promoting project (7 likes, 1 repost) | 7 | 1 | |
| | 18 June, Twitter, promoting project, (11 likes, 5 reposts) - NHMU | 11 | 5 | |
| | 24 June, Monarchs of Bridgerland, virtual present. CB | na | na | #VALUE! |
| | 26 June, Twitter, promoting project, inc Wyoming, (6 likes, 1 repost) - NHMU | 6 | 1 | |
| | 30 June, Twitter, promoting project (3 likes, 3 reposts) - NHMU | 3 | 3 | |
| | 6 July, Twitter, promoting project (9 likes, 3 reposts) - NHMU | 9 | 3 | |
| | | | | 0 |
| 2021 | | | | 0 |
| | 27 April, FB, promoted with other CS projects, (3 rxns) - NHMU | 3 | na | 3 |
| | 28 April, FB, promoted with other CS projects, (1 comment, 21 shares, 129 rxns) | 129 | 21 | 150 |
| | 3 Jun, FB, promoting blog describing project (8 comments, 24 shares, 151 rxns) - NHMU | 151 | 24 | 175 |
| | 9 June, FB, promoting partnership with New Mexico (3 comments, 5 shares, 88 rxns) - NHMU | 88 | 5 | 93 |
| | 9 Oct Park City Library, present.- CB | na | na | na |
| | | | | 0 |
| 2022 | | | | 0 |
| | 7 June, FB, promoting Uintah Co Library talk - NHMU (12 rxns) | 12 | na | 12 |
| | 13 Apr NHMU, virtual present. - CB | na | na | na |
| | 8 June, Twitter, promoting presentation at Uintah library (2 likes, 1 repost) - NHMU | 2 | 1 | |
| | 10 Jun, FB, project promoted with Bugfest & Uintah library talk (4 rxns) - NHMU | 4 | na | 4 |
| | 10 Jun, Twitter, presentation at Uintah County Library promoted (16 likes, 5 reposts) | 16 | 5 | |
| | 14 Jun, FB, mentioned in promotion video of general ent questions answered (3 rxns)- NHMU | 3 | na | 3 |
| | 15 Jun Uintah Co Library, present.- CB | na | na | na |
| | 15 Jun, Twitter, promoting Uintah Co library talk - (3 likes) NHMU | 3 | 0 | |
| | 17 Jun FB, mentioned with Bugfest promo, (3 rxns) - NHMU | 3 | na | 3 |
| | 17 Jun, Twitter, promoted project, (2 likes) - NHMU | 2 | 0 | |
| | 22 Jun, Twitter, promoting project (5 likes, 1 repost) - NHMU | 5 | 1 | |
| | 23 Jun, Twitter, promoting project (2 likes, 1 repost) - NHMU | 2 | 1 | |
| | 29 Jun, Twitter, sharing NHMU Youtube video of Charleston fireflies (4 likes) - NHMU | 4 | 0 | |
| 2023 | | | | |
| | Jun 12, Twitter promoting project (6 likes, 1 repost) - NHMU | 6 | 1 | |
| | Jun 13, sharing abc4 article, Nibley fireflies (11 rxns, 3 shares) - NHMU | 11 | 3 | |
| | Jun 28, FB promoting project (12 rxns, 3 shares) - Bear River Migratory Bird Refuge | 12 | 3 | |
| | July 17, Twitter, promoting project (3 likes) - NHMU | 3 | 0 | |
| | July 17, FB, promoting project (64 rxns, 24 shares) | 64 | 24 | |
| | Oct 30, Twitter, promoting project (3 likes) - NHMU | 3 | 0 | |
| | Oct 30, FB, sharing PBS Terra video (9 reactions, 1 share) | 9 | 1 | |
| | | **7046** | **1760** | 8806 |
